# Unveiling the Diversity of Periphytic Cyanobacteria (Cyanophyceae) from Tropical Mangroves in Penang, Malaysia

**DOI:** 10.21315/tlsr2023.34.3.4

**Published:** 2023-09-30

**Authors:** Nur Afiqah Abdul Rahim, Faradina Merican Mohd Sidik Merican, Ranina Radzi, Wan Maznah Wan Omar, Siti Azizah Mohd Nor, Paul Broady, Peter Convey

**Affiliations:** 1 School of Biological Sciences, Universiti Sains Malaysia, 11800 USM Pulau Pinang, Malaysia; 2 Institute of Marine Biotechnology, Universiti Malaysia Terengganu, 21300 Kuala Terengganu, Terengganu, Malaysia; 3 School of Biological Sciences, University of Canterbury, 20 Kirkwood Avenue, Upper Riccarton, Christchurch 8041, New Zealand; 4 British Antarctic Survey, NERC, High Cross, Madingley Road, Cambridge CB3 0ET, United Kingdom

**Keywords:** Cyanobacteria, Morphospecies, Phenotypic Diversity, Tropical Mangrove, Cyanobacteria, Morfospesies, Kepelbagaian Fenotopik, Bakau Tropika

## Abstract

Cyanobacteria are one of the most important groups of photoautotrophic organisms, contributing to carbon and nitrogen fixation in mangroves worldwide. They also play an important role in soil retention and stabilisation and contribute to high plant productivity through their secretion of plant growth-promoting substances. However, their diversity and distribution in Malaysian mangrove ecosystems have yet to be studied in detail, despite Malaysia hosting a significant element of remaining mangroves globally. In a floristic survey conducted in Penang, peninsular Malaysia, 33 morphospecies of periphytic cyanobacteria were identified and described for the first time from a mangrove ecosystem in Malaysia. Sixteen genera, comprising *Aphanocapsa, Chroococcus, Chroococcidiopsis, Cyanobacterium, Desmonostoc, Geitlerinema, Leptolyngbya, Lyngbya, Microcystis, Myxosarcina, Oscillatoria, Phormidium, Pseudanabaena, Spirulina*, *Trichocoleus* and *Xenococcus*, were obtained from field material growing on diverse natural and artificial substrata. Oscillatoriales was the dominant order with *Phormidium* the dominant genus at nine of the 15 sampling sites examined. Three of the morphospecies, *Aphanocapsa* cf. *concharum, Xenococcus* cf. *pallidus* and *Oscillatoria pseudocurviceps*, are rare and poorly known morphospecies worldwide. *Chroococcus minutus, Phormidium uncinatum, P. amphigranulata*, and some species of Oscillatoriales are considered as pollution indicator species. This study provides important baseline information for further investigation of the cyanobacterial microflora present in other mangrove areas around Malaysia. A complete checklist will enhance understanding of their ecological role and the potential for benefits arising from useful secondary metabolites or threats via toxin production to the ecosystem.

## Abstrak

Cyanobacteria adalah salah satu kumpulan organisma fotoautotropik yang paling penting yang menyumbang kepada penetapan karbon dan nitrogen dalam paya bakau di seluruh dunia. Mereka juga memainkan peranan yang penting dalam pengekalan dan penstabilan tanah dan menyumbang kepada produktiviti tumbuhan yang tinggi melalui rembesan bahan penggalak pertumbuhan tumbuhan. Walau bagaimanapun, kepelbagaian dan taburannya dalam ekosistem paya bakau Malaysia masih belum dikaji secara terperinci, walaupun Malaysia menjadi tuan rumah elemen penting bagi baki bakau di peringkat global. Dalam tinjauan floristik yang dijalankan di Pulau Pinang, Semenanjung Malaysia, 33 morfospesies cyanobacteria perifit telah dikenal pasti dan diterangkan buat kali pertama daripada ekosistem bakau di Malaysia. Sebanyak 16 genera, yang terdiri daripada *Aphanocapsa, Chroococcus, Chroococcidiopsis, Cyanobacterium, Desmonostoc, Geitlerinema, Leptolyngbya, Lyngbya, Microcystis, Myxosarcina, Oscillatoria, Phormidium, Pseudanabaena, Spirulina, Trichocoleus* dan *Xenococcus*, yang tumbuh secara semula jadi diperolehi daripada pelbagai bahan tumbuh-tumbuhan semula jadi dan tiruan. *Oscillatoriales* ialah susunan dominan dengan *Phormidium* merupakan genus dominan di 9 daripada 15 tapak persampelan yang diperiksa. Tiga daripada morfospesies, *Aphanocapsa* cf. *concharum*, *Xenococcus* cf. *pallidus* dan *Oscillatoria pseudocurviceps*, adalah morfospesies yang jarang dan kurang dikenali di seluruh dunia. *Chroococcus minutus, Phormidium uncinatum, P. amphigranulata*, dan beberapa spesies *Oscillatoriales* dianggap sebagai spesies penunjuk pencemaran. Kajian ini menyediakan maklumat asas yang penting untuk penyiasatan lanjut tentang mikroflora *cyanobacterial* yang terdapat di kawasan bakau lain di sekitar Malaysia. Senarai semak yang lengkap akan meningkatkan pemahaman tentang peranan ekologi mereka dan potensi manfaat yang timbul daripada metabolit sekunder yang berguna atau ancaman melalui pengeluaran toksin kepada ekosistem.

**Highlights**

- Thirty-three morphospecies of periphytic cyanobacteria have been identified in a mangrove ecosystem in Malaysia.
- This is the first record of cyanobacterial diversity in a Malaysian mangrove.
- This is also the first checklist for Malaysian mangroves that will help identify possible alternative sources of secondary metabolites and threats in the ecosystem.

## INTRODUCTION

Mangroves are marine coastal ecosystems that constitute a transitional forest between the coast and the mainland. Mangroves are highly productive ecosystems and create unique niches for a diversity of plants and animals as well as providing nursery grounds for many benthic and pelagic marine organisms (Yabuki 2004; Alongi 2005; Sundararaman *et al*. 2007; Rigonato *et al*. 2013). Malaysia is one of six countries (the others being Indonesia, Australia, Brazil, Mexico and Nigeria) which together host 50.5% of the world’s mangroves (Alvarenga *et al*. 2015). Mangroves are a common feature in coastal areas of Malaysia, with the largest areal contribution particularly along the north-east coast of Sabah. In Sarawak, most mangroves are located in the deltas of the Sarawak, Rejang and Trusan-Lawas Rivers [Food and Agriculture Organisation (FAO) 2014]. Mangroves sustain and support production, income and employment in coastal fisheries.

Most published research on mangroves in Malaysia has focused on faunal studies, generally on the diversity of fish and their juveniles (Hoque *et al*. 2015; Abu Hena *et al*. 2017; Azmir *et al*. 2017). Other studies have investigated molluscs (Singh & Baharin 2016; Ismail *et al*. 2017; Vaezzadeh *et al*. 2017), horseshoe crabs (Noor Jawahir *et al*. 2017), mud crabs (Sharif *et al*. 2016) and diverse fauna (Zahidin *et al*. 2016) and food webs (Le *et al*. 2017). Studies on flora and mangrove distribution (Hamzah *et al*. 2009; Shah *et al*. 2016), sediment analyses (Mokhtari *et al*. 2016; Pazi et al. 2016; Atwood *et al*. 2017; Bakrin Sofawi *et al*. 2017; Mustapha *et al*. 2017), organic matter (Hemati *et al*. 2017) and heavy metals (Baruddin *et al*. 2017) have also been carried out. In the field of microbiology, studies of the diversity and properties of Actinobacteria (Azman *et al*. 2016, 2017; Ser *et al*. 2016, 2017; Zainal *et al*. 2016; Law *et al*. 2017; Tan *et al*. 2017), Proteobacteria (Moh *et al*. 2017) and Firmicutes (Auta *et al*. 2017) have been conducted. Studies have also addressed the diversity of macroalgae (Billah *et al*. 2016; Md Isa *et al*. 2017) and microalgae (specifically diatoms) (Tan *et al*. 2016; Majewska *et al*. 2017). A limited number of bioprospecting studies of fungi have also been carried out (Seydametova *et al*. 2015).

Cyanobacteria are one of the earliest organisms to have evolved on earth and may have existed for 3.5 billion years (Komárek 2016). They play a major role in oxygen production and nutrient cycling, with some members of the group also capable of fixing atmospheric nitrogen (Gehringer & Wannicke 2014). Among the highly diverse microbial communities in mangrove ecosystems, cyanobacteria are one of the most commonly noted organisms, adapted to highly unstable environmental conditions (Alongi 2005; Nedumaran *et al*. 2008; Rigonato *et al*. 2013). They are one of the main primary producer groups that support marine fauna and fisheries in mangrove ecosystems (Essien *et al*. 2008), carrying out the same photosynthetic function as do eukaryotic algae. The organic material produced by these organisms is the foundation of the entire food web in these ecosystems (Dadheech *et al*. 2013). Cyanobacterial communities can be observed developing on a variety of surfaces in mangroves, such as sediments, roots, leaves and branches (Rigonato *et al*. 2013).

Cyanobacteria vary morphologically and physiologically in response to different environmental conditions, making them reliable environmental indicators in the mangrove ecosystem (Chaurasia 2015). Morphological characteristics can provide valuable information about the nutrient status of a site. The presence of well-developed multicellular hyaline hairs in many filamentous forms is a response to phosphorus limitation (Chaurasia 2015). A higher number of heterocytes in trichomes is an indicator of water lacking combined nitrogen compared to other nutrients, especially phosphate (Chaurasia 2015). Changes have also been demonstrated in the composition of the cyanobacterial community as a function of water quality. An increase in abundance of heterocytic cyanobacteria such as *Calothrix, Scytonema, Nostoc* and *Rivularia* has been observed in response to low nitrate concentrations, while mass growth of Oscillatoriales species can be associated with eutrophication (Mateo *et al*. 2015). Shifts in species diversity, along with changes in morphological characteristics and photosynthetic behaviour, are reliable indicators that can be utilised to assess environmental changes (Wan Jusoh *et al*. 2020).

Some cyanobacteria also have proven ability to produce useful bioactive natural compounds, which may have antifungal, antibacterial, antiviral and protease inhibition activities (Shishido *et al*. 2015; Briand *et al*. 2016). However, some species can also have strongly detrimental effects on the environment, humans, animals and other organisms through their ability to produce toxins under certain environmental condition (Lopes & Vasconcelos 2011; Neilan *et al*. 2013; Silva *et al*. 2014; Briand *et al*. 2016). Non-ribosomal peptide synthetase (NRPS) and polyketide synthase (PKS) enzymes (Silva *et al*. 2014) are involved in the synthesis of most compounds produced by cyanobacteria. However, to further develop the potential of applied research on these organisms, their diversity and distribution must first be understood.

Amongst the countries hosting the world’s mangroves, a significant amount of research on cyanobacteria has been conducted in Brazil alone (Nogueira & Ferreira-Correia 2001; Rigonato *et al*. 2012; Alvarenga *et al*. 2015). Although India makes a relatively small contribution to global mangrove extent, it has been an active source of research on mangrove cyanobacteria (Sakthivel & Kathiresan 2013; Diana & Papiya 2014; Ray *et al*. 2014).

The study of cyanobacterial biodiversity and distribution in brackish environments has been neglected worldwide (Ram & Paul 2021). To our knowledge, studies of cyanobacterial diversity in Malaysia have been conducted solely in freshwater environments and aquaculture ponds, and then only with identification to the genus level (Mohd. Nasarudin & Ruhana 2007; 2011a; 2011b; Sinang *et al*. 2015; Sinden & Sinang 2015). The existence and diversity of cyanobacteria colonising Malaysian mangroves requires recognition, identification of their potential as bioindicators or as producers of useful bioactive compounds, and of threats to the ecosystem (including associated and dependent human populations). Here we present the first extensive study of cyanobacterial diversity in a Malaysian mangrove ecosystem.

## MATERIALS AND METHODS

### Sample Collection

**Figure 1:**
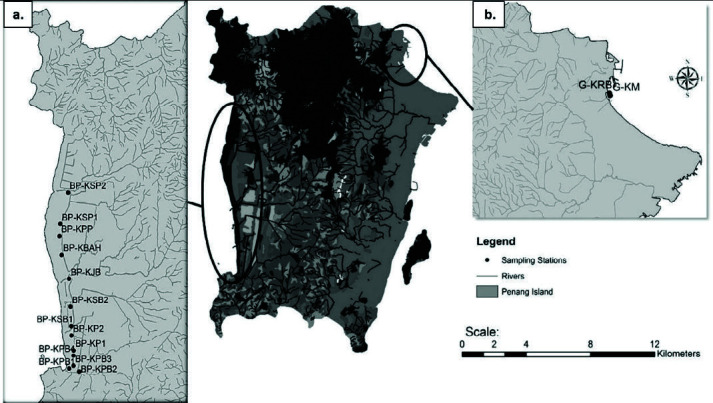
Map showing the study sites at Penang Island, Malaysia (centre). (a) Balik Pulau and (b) Gurney. *Source*: Derived from ArcGis.

A floristic survey was conducted at all accessible mangrove locations along Balik Pulau, located in the south-west of Penang Island, and Gurney, located in the north-east of Penang Island (Fig. 1) between December 2014 and October 2015. Fifteen mangrove sampling sites, including 13 from Balik Pulau and two from Gurney, were examined. Summary descriptions of these sampling sites are presented in Table 1.

**Figure 2:**
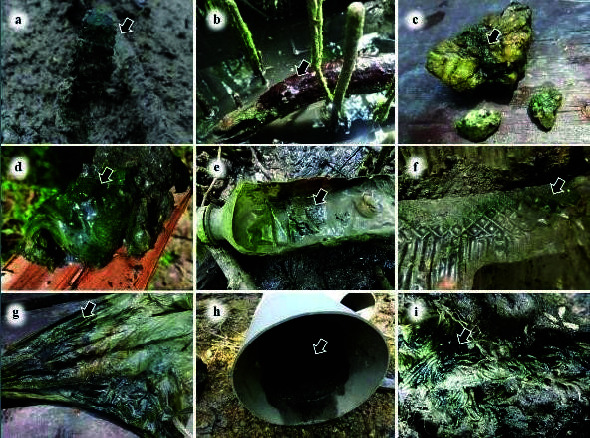
A variety of periphytic cyanobacteria growing on different substrates. (a) bluegreen mats on a pneumatophore (arrow); (b) brown mats on a rotting tree branch (arrow); (c) olive-green crust on rocks (arrow); (d) blue-green mats on sediments (arrow); (e) blue-green crust inside a plastic bottle (arrow); (f) blue-green crust inside a plastic food container (arrow); (g) dark blue-green crust inside a plastic bag (arrow); (h) dark blue-green mats on sediments inside PVC pipe (arrow); (i) dark blue-green mats on linen rope (arrow).

All visible cyanobacterial growths were sampled from different natural and artificial substrates (Fig. 2). The natural substrates included pneumatophores of mangrove trees, rotting tree branches, rock and sediment, while the artificial substrates included plastic bottles, plastic food containers, plastic bags, PVC pipe and linen rope. Each site was examined and samples of visible mats, gelatinous colonies or crust were collected. Crusts from the substrate were removed by scraping the surface using a spatula while mats and gelatinous colonies were removed by hand. Samples were kept in 60 mL polycarbonate screw-top containers and transported to the laboratory for further analysis.

### Microscopic Evaluation

Slides containing fresh material mounted in habitat water were observed under a compound microscope (Olympus BX53) at 100–2,000× magnification. Diacritical characteristics for morphological identification included: vegetative cell width and length, and those of other specialised cells if present; heterocytes and akinetes were recorded and 30 random measurements were taken. Other characteristics of the samples including cell shape and colour, the presence of granules, constriction of cross walls and the structure of apical cells were recorded. Illustrations were made with the aid of a *camera lucida*. Identifications were made to the lowest taxonomic level possible following Komárek and Anagnostidis (1998; 1999; 2005) and Komárek (2013a; 2013b). The abbreviation “cf.” (Latin *confertim*: to compare with) was used when uncertainty existed, indicating ambiguity between the identified specimen and closely similar morphospecies in the identification key. The high dependence on temperate region keys in identifying specimens can potentially lead to the loss of information on the possibility of a new variety of a given species or even of a novel species. We have taken a conservative approach in listing all the differences between the strains to ensure information on morphospecies that might be useful in confirming variant forms or novel species will be available to future researchers.

### Culture Establishment

All samples were cultivated and maintained in 1% agarised liquid BG-11 or BG-110 (lacking nitrogen) media (Rippka *et al*. 1979) supplemented with 0.1 μg vitamin B12, artificial seawater adjusted to 6 ppt salinity and 100 μg/mL cycloheximide (Bolch & Blackburn 1996). Samples were incubated at 25°C and 12:12 h light:dark under a white fluorescent lamp (±27.03 mmol m^−2^ s^−1^).

## RESULTS

Thirty-three morphospecies of cyanobacteria including 3 Chroococcales, 3 Chroococcidiopsidales, 3 Nostocales, 13 Oscillatoriales, 3 Pleurocapsales, 3 Spirulinales and 7 Synechococcales were identified from the Malaysian mangrove ecosystems examined. Each morphospecies encountered was rated as present (+) or dominant (++) (Table 2). Thirty-one of the morphospecies were described from field specimens. Of these, 11 were successfully grown in culture – eight morphospecies in unialgal and three in mixed cultures. Two morphospecies were only observed in culture.

### Family Chroococcaceae

**Figure 3:**
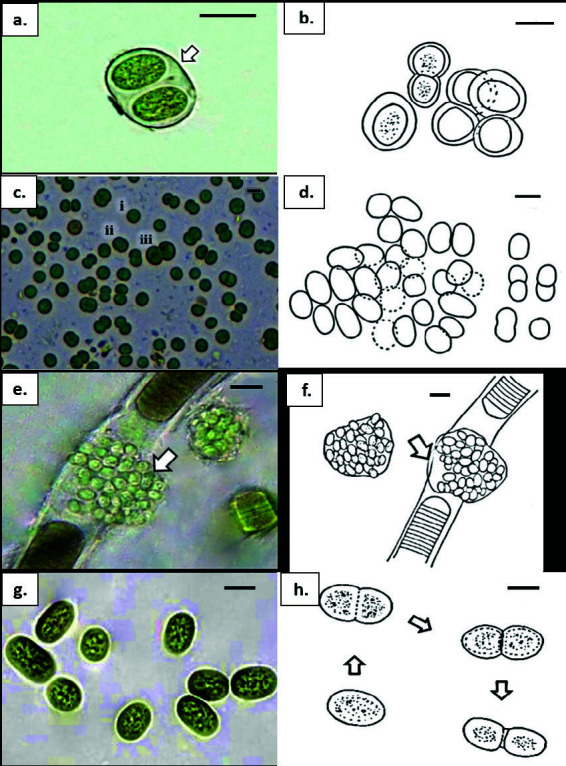
*Chroococcus minutus***:** (a) thick, firm and colourless mucilaginous envelope (arrow); (b) cells in colony undergo cell division. *Microcystis halophilia*: (c) (i–iii), bright blue-green, free-living dividing cells from cultures; (d) cell division occurs perpendicular to the longer axis. *Aphanocapsa* cf. *concharum*: (e) colonies in thin, firm mucilaginous sheath of *Lyngbya* sp. (arrow); (f) spherical to oval cells in communal sheath (arrow). *Cyanobacterium* cf. *cedrorum*: (g) dark olive-green, oval to rod-shaped cells with homogenous content without individual sheath; (h) Stages of cell division. Scale bars: a–h = 5 μm.

***Chroococcus minutus*** (Kützing) Nägeli 1849 (Figs. 3a and 3b)

Komárek and Anagnostidis (1998): p. 296, figure 391 (p. 297)

Description: Field specimens epiphytic on rotting tree branch (Fig. 2b) forming blackish, slimy mat. Cells solitary or two-cell colonies (Fig. 3a), pale blue-green or olive green, spherical, oval with homogeneous content, 5.0 μm–6.5 μm diameter. Reproduction by simple binary fission (Fig. 3b). Sheath individual, thick, hyaline.

Ecology: Sites BP-KPB3, BP-KP1 and G-KM.

Notes: *C. minutus* is cosmopolitan with previous records from the brackish milkfish ponds of Marakei and Nikunau Atolls, Republic of Kiribati, in the central Pacific (Tebano 2008). Recorded by John *et al*. (2002), *C. minutus* occurred mostly in shallow water bodies and usually with other macrophytes.

### Family Microcystaceae

***Microcystis halophilia*** (Hansgirg) Komárek & Anagnostidis 1995 (Figs. 3c and 3d)

Komárek and Anagnostidis (1998): p. 159

Description: Field specimens periphytic on linen rope (Fig. 2i). Cells densely arranged, bright blue-green or yellowish-green, spherical or oval without homogeneous content, 3.0 μm–5.0 μm diameter, distinctly longer before cell division by binary fission. Sheath communal, thin, hyaline.

Ecology: Site BP-KJB

Notes: Previously recorded from India on mangrove plants (Selvakumar & Sundararaman 2001) and on soils (Silambarasan *et al*. 2012).

### Family Merismopediaceae

***Aphanocapsa* cf. *concharum*** Hansgirg 1890 (Figs. 3e and 3f)

Komárek and Anagnostidis (1998): p. 154, Fig. 173 (p. 154)

Description: Field specimens periphytic in plastic bag (Fig. 2g). Also occurred on the sheath of other cyanobacteria. Cells densely arranged, pale blue-green, spherical or spherical-ellipsoidal without homogeneous content, 1.5μm–2.0 μm diameter, sometimes longer up to 3.0 μm before cell division. Reproduction by simple binary fission in two perpendicular planes. Sheath absent.

Ecology: Site BP-KPB1

Notes: Specimens differ from previously described *A. concharum* (Komárek & Anagnostidis 1998) in having slightly longer cells (1.0 μm–1.6 μm). The morphospecies was previously recorded from the marine environment as epiphytic on mollusc shells and algae (Komárek & Anagnostidis 1998). The presence of this morphospecies in brackish water has been less frequently documented.

### Family Cyanobacteriaceae

***Cyanobacterium* cf. *cedrorum*** (Sauvageau) Komárek *et al*. 1999 (Figs. 3g and 3h)

Komárek and Anagnostidis (1998): p. 46, Fig. 3 (p. 46)

Description: Field specimens epiphytic on rotting tree branch (Fig. 2b). Cells solitary or two-cell colonies, dark olive-green to brownish, oval, almost elongated to rod-shaped with rounded ends and homogeneous content, 5.0–8.8 μm × 3.8–5.0 μm. Reproduction by simple binary fission in one direction, perpendicular to the long axis. Sheath absent.

Ecology: Sites BP-KPB1, BP-KPB3 and BP-KP

Notes: Cell dimensions are within the range described for *C. cedrorum* (Komárek & Anagnostidis 1998), a species previously recorded from subaerophytic habitats on tree branches from warm areas including the northern temperate zone, subtropical and tropical countries (Komárek & Anagnostidis 1998). Molecular and cytomorphological approaches are required for further taxonomic evaluation of this morphospecies.

### Family Chroococcidiopsidaceae

**Figure 4:**
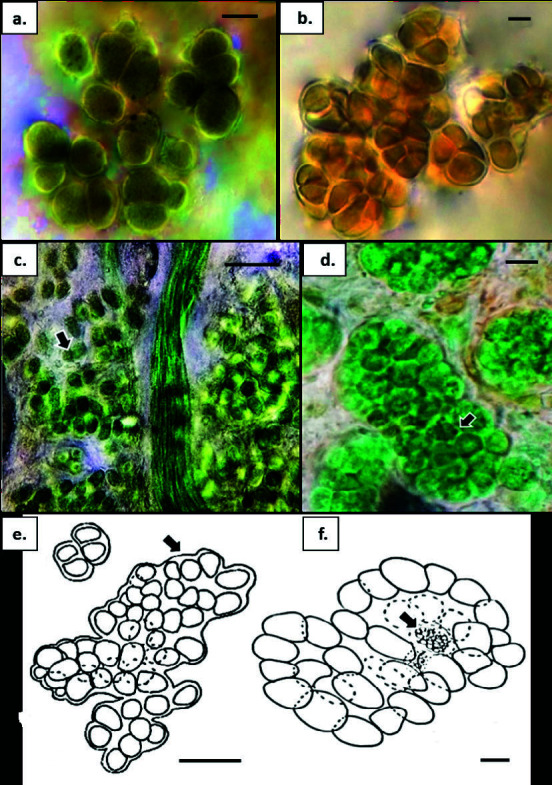
(a) Chroococcidiopsis cf. thermalis. bright blue-green, irregular flat colonies. (b) Myxosarcina cf. gloeocapsoides. Olive-green to brownish, densely packed cells. (c) & (e) Xenococcus cf. pallidus. bright blue-green, irregular cells forming colonies among Trichocoleus tenerrimus; thin communal sheath. (d) & (f) Xenococcus cf. schousboei. bright blue-green cells packed densely in colonies, forming communal mucilaginous envelope with visible spherical to irregular shape of baeocytes (arrow). Scale bars: a–f = 5 μm.

***Chroococcidiopsis cf. thermalis*** Geitler 1933 (Fig. 4a)

Komárek and Anagnostidis (1998): p. 421, Figs. 549 and 550 (p. 421)

Description: Field specimens periphytic in plastic bottles (Fig. 2e). Cells solitary or up to four-cell colonies, dark olive-green, spherical, irregularly rounded without homogeneous content, 6.5 μm–8.2 μm diameter. Reproduction irregularly by binary fission. Baeocyte not observed. Sheath individual, thin, hyaline.

Ecology: Sites BP-KPB1 and BP-KPB3

Notes: Cells spherical or irregular in shape, clustering into non-polarized agglomerations forming rounded colonies separated by short distances, features suggesting placement in *Chroococcidiopsis* (Donner 2013). Specimens differ from *C. thermalis* (Komárek & Anagnostidis 1998) in smaller cell dimension (9.0 μm–10.0 μm) and the absence of baeocytes. Previously described as periphytic on stones in mineral and thermal springs, mainly from tropical countries (Komárek & Anagnostidis 1998) and in brackish water, features indicating strong tolerance to stressful environmental conditions (John & Lynn 2014).

### Family Hyellaceae

***Myxosarcina cf. gloeocapsoides*** (Setchell et Gardner)

Komárek and Anagnostidis 1995 (Fig. 4b)

Komárek and Anagnostidis (1998): p. 427, Fig. 361e (p. 276), Fig. 556 (p. 424)

Description: Field specimens epilithic on rock (Fig. 2c) and periphytic in plastic bag (Fig. 2g). Colonies packet–like, one or two layers of cells, olive-green to brownish, slightly oval, irregular without homogeneous content, 3.0–5.5 μm × 4.0–6.5 μm. No nanocyte observed. Sheath communal, thin, hyaline.

Ecology: Sites BP-KPB1 and G-KM

Notes: Specimens differ from *M. gloeocapsoides* described by Komárek and Anagnostidis (1998) through having smaller cell diameter, 4.0 μm–8.0 μm. The morphospecies was previously recorded from a salt marsh in California and is known to occur on coastal rocks in the Mediterranean (Komárek & Anagnostidis 1998). Tika Khusnul *et al*. (2014) recorded *Myxosarcina* sp. as epiphyte on an *Avicennia marina* pneumatophore in a mangrove ecosystem in Indonesia.

### Family Xenococcaceae

***Xenococcus* cf. *pallidus*** Hansgirg Komárek & Anagnostidis 1995 (Figs. 4c & 4e) Komárek and Anagnostidis (1998): p. 429, Fig. 560 (p. 424)

Description: Field specimens epiphytic on pneumatophores (Fig. 2a) and epipelic on sediments (Fig. 2d). Cells densely aggregated, bright blue–green, rounded or irregular without homogeneous content, 2.8 μm–3.5 μm diameter, epiphytic to other cyanobacteria. No baeocyte observed. Sheath communal, thin, yellowish.

Ecology: Sites BP-KPB1 and BP-KSB2

Notes: Colonies forming irregular cells aggregated into one layer on the substrate suggest placement in *Xenococcus pallidus*. Specimens slightly shorter, 3.0 μm–5.0 μm, than *X. pallidus* described by Komárek and Anagnostidis (1998). Morphospecies was previously recorded from marine environment epiphytic on seaweeds (Komárek & Anagnostidis 1998).

***Xenococcus* cf. *schousboei*** Thuret 1880 (Figs. 4d & 4f)

Komárek and Anagnostidis (1998): p. 430, Fig. 562 (p. 431)

Description: Field specimens epiphytic on rotting tree branches (Fig. 2b). Cells densely arranged, bright blue-green, older cells yellowish, and spherical to almost cylindrical with homogeneous content, 4.0 μm–5.5 μm diameter. Reproduction by baeocyte division. Baeocytes spherical to irregular in shape, 1.0 μm–2.0 μm diameter. Sheath communal, thin, hyaline.

Ecology: Sites BP-KPB2, BP-KP and G-KM

Notes: Cell dimensions consistent with the description of Komárek and Anagnostidis (1998), although this did not include baeocytes. The morphospecies has been recorded as epiphytic on seaweeds in marine habitats (Komárek & Anagnostidis 1998), and from Northern Cyprus in the marine benthos (Ulcay *et al*. 2015).

### Family Nostocaceae

**Figure 5:**
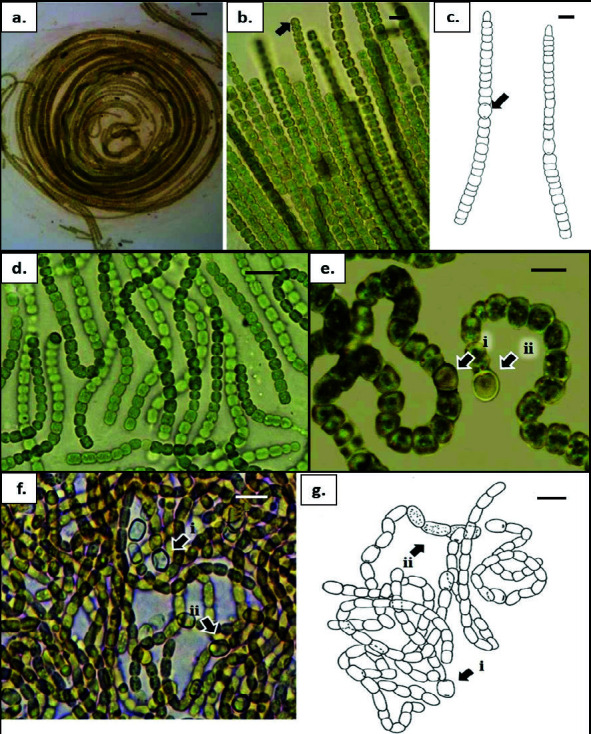
(a)–(c) Anabaena sp. dark olive green to brownish colonies with trichome entangled forming mat; (b) barrel-shaped vegetative cells with rounded apical cell (arrow); (c) slightly pointed at terminal trichome with intercalary heterocyst (arrow); (d) & (e) Desmonostoc muscorum; (d) barrel-shaped to cylindrical coiled trichome; (e) heterocytes positioned at intercalary [(arrow (i)] and terminal [(arrow ii)] of the trichome; (f) & (g) Nostoc sp. (f) barrel-shaped to cylindrical, coiled and longer than wide trichome; (f) (arrow i); (g) (arrow i), terminal positioned heterocyte; (g) (arrow ii), intercalary positioned heterocytes. (g) (arrow ii) old cells forming akinete. Scale bars: a–g = 5 μm.

***Anabaena* sp**. (Figs. 5a–5c)

Komárek and Anagnostidis (2013): p. 790

Description: Trichome entangled, pale blue-green to olive-green, isopolar. Cells barrel-shaped to cylindrical, longer than wide, 3.8 μm–6.3 μm wide, 5.5 μm–7.5 μm long. Apical cell rounded to slightly pointed. Heterocytes develop intercalary positions, spherical to oval shaped, 3.8–5.0 μm × 6.0–8.5 μm. No akinete observed.

Ecology: Specimen did not form macroscopic growth in the field and was only observed in cultures.

Notes: Specimens recorded only from cultures in agar plate and liquid media. Trichomes in mat forming flat macroscopic, entangled, colonies suggest placement in *Anabaena* sp. Akinete morphology is the main characteristic to identify this species (Komárek 2013a). This feature serves as a dormant cell in unfavourable conditions and their absence in the cultured strain has been recorded previously (Komárek 2013b), but hinders the identification of this strain.

***Desmonostoc muscorum*** Hrouzek et Ventura 2013 (Figs. 5d & 5e)

Komárek and Anagnostidis (2013): p. 1016, Figs. 1281 and 1327 (p. 987 & p. 1015)

Description: Field specimens epiphytic on rotting tree branches (Fig. 2b). Trichomes blue-green to olive-green, entangled forming coiled microscopic colonies amongst other cyanobacteria, isopolar. Cells barrel-shaped to cylindrical, shorter than wide or nearly isodiametric, 3.5 μm–6.0 μm wide, 3.0 μm–5.2 μm long. Apical cells rounded, not attenuated. Heterocytes develop in both terminal and intercalary positions, spherical to almost barrel-shaped, 4.4–5.0 μm × 4.0–4.8 μm. No akinete observed.

Ecology: site BP-KPB3

Notes: Recently revised from *Nostoc muscorum* Agardh ex Bornet et Flahault 1888. Trichome width and length, and size of heterocytes, consistent with the description of *Desmonostoc muscorum* (Komárek & Anagnostidis 2013). Previously recorded as cosmopolitan, present in wet soils, among mosses, rocks, from lowlands to mountains, thermal springs and in saline localities (Komárek & Anagnostidis 2013).

***Nostoc* sp**. (Figs. 5f & 5g)

Komárek and Anagnostidis (2013): p. 953

Description: Colonies on agarised and in liquid media forming dark olive-green to brownish-green crust. Trichome bright blue-green to dark olive-green, isopolar. Cells barrel-shaped to cylindrical, longer than wide, 2.0 μm–2.5 μm wide, 3.8–7.5 μm long. Apical cell rounded. Heterocytes develop in both terminal and intercalary positions, spherical to cylindrical shaped, 3.8–5.0 μm × 5.0–6.4 μm. Akinetes oval, slightly larger than vegetative cells, 3.8–5.0 μm × 5.0–7.5 μm.

Ecology: Specimen did not form macroscopic growth in the field and was only observed in cultures.

Notes: Specimens recorded only from cultures in agar plate and liquid media. Trichomes aggregated, densely entangled in colonies suggest placement in *Nostoc*. Specimens similar morphologically to *Nostoc passerinianum* described by Komárek and Anagnostidis (2013) in colony formation and cell shape, but differ ecologically, with *N. passerinianum* reported on wet soil mainly from Europe and with cell dimensions 5.0–7.0 μm × 4.0 μm, heterocytes ±5.0 μm and akinetes, 6.0–8.0 μm × 5.0–6.0 μm.

### Family Oscillatoriaceae

**Figure 6:**
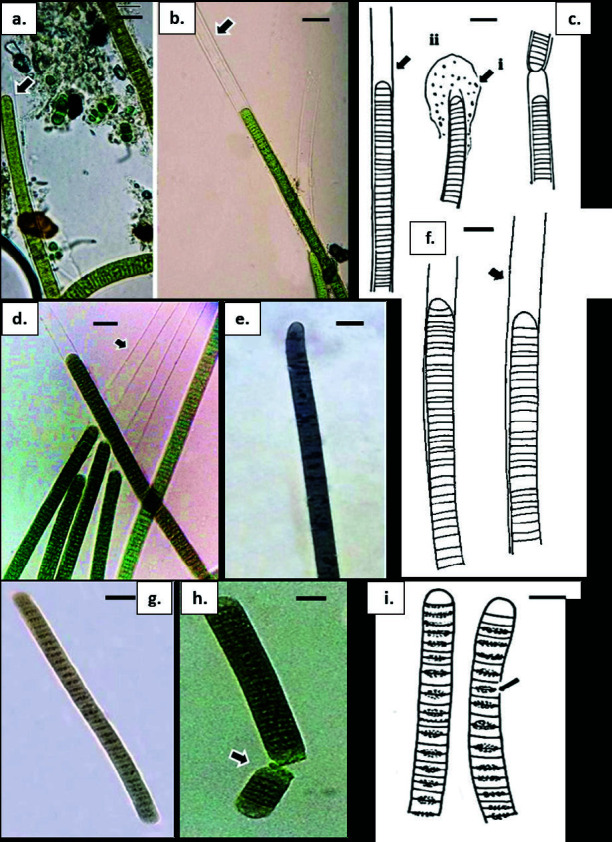
(a–c) Lyngbya cf. aestuarii; (a) filaments olive-green in colour with rounded apical cell (arrow); (b & c) trichome distinctly shorter than wide with colourless, hyaline, firm sheath [(arrow (ii)] with some of it attached to organic matter [(arrow(i)]. (d & f) Lyngbya cf. salina; (d & f) filament morphology with shorter than wide cells, rounded apical cells and fine hyaline sheath (arrow); (e) Oscillatoria pseudocurviceps. (e) olive-green to greyish trichome with distinctly shorter than wide cells, conically rounded apical cell with granulated cell content; (g–i) Oscillatoria rupicola; (g) trichomes olive-green to bright blue-green, straight or slightly curved with shorter than wide cells and rounded apical cell; (h) hormogonia division by necridic cell (arrow); (i) granulated cross walls (arrow). Scale bars: a–d & f = 10 μm; e = 15 μm; g–i = 5 μm.

***Lyngbya* cf. *aestuarii*** Liebman ex Gomont (1892) (Figs. 6a–6c)

Komárek and Anagnostidis (2005): p. 621, Figs. 947 and 948 (p. 622 and p. 624)

**Description:** Field specimens epipelic on sediments (Fig. 2d), periphytic in plastic bag (Fig. 2g) and epilithic on rock (Fig. 2c). Filaments olive-green, entangled among other microalgae, straight, isopolar, 8.0 μm–9.0 μm wide. Cells cylindrical, distinctly shorter than wide, 6.8–8.2 μm wide, 1.2–3.2 μm long, not constricted at cross wall. Apical cells rounded, not attenuated, with no calyptra. Sheath thin, hyaline.

Ecology: Sites BP-KSB2 and BP-KSP2

Notes: Specimens differ in slightly narrower [10.0–21.0 (24.0)] μm and slightly shorter [2.0–5.6(6.0)] μm trichome from *L. aestuarii* described by Komárek and Anagnostidis (2005). Previously recorded as halophilic, benthic and periphytic on rocks, sandy bottoms and loam as well as in brackish waters (Komárek & Anagnostidis 2005). Bagmi *et al*. (2007) reported *L. aestuarii* in mat formation associated in a subtropical mangrove with exposure to solar radiation.

***Lyngbya* cf. *salina*** (Kützing ex Starmach) 1966 (Figs. 6d & 6f)

Komárek and Anagnostidis (2005): p. 618, Fig. 938 (p. 617)

Description: Field specimens epiphytic on rotting tree branch (Fig. 2b). Filaments bright blue-green, clustered, long, straight or slightly bent, isopolar. Cells cylindrical, distinctly shorter than wide, 7.5 μm–10.0 μm wide, 1.0 μm–3.8 μm long, not constricted at cross walls. Apical cells rounded, not attenuated, with no calyptra. Sheath thin, hyaline.

Ecology: Sites BP-KPB3 and BP-KP1

Notes: The presence of obligatory firm sheaths and the short cells suggests placement in *Lyngbya* (Komárek & Anagnostidis 2005). Specimens differ from previously described *L. salina* in having slightly narrower [8–14 (15) μm] and shorter (2–2.5 μm) cells. The morphospecies was previously recorded from saline water mainly in coastal swamps in the temperate region (Wehr *et al*. 2015).

***Oscillatoria cf. pseudocurviceps*** Welsh (1965) (Fig. 6e)

Komárek and Anagnostidis (2008): p. 601, Fig. 905 (p. 600)

Description: Field specimens epipelic (Fig. 2d). Trichomes olive-green to greyish, straight, actively motile, isopolar. Cells cylindrical, distinctly shorter than wide, 13.8 μm–15.0 μm wide, 5.0 μm–6.5 μm long, not constricted at cross walls. Cell content granulated. Apical cells rounded or slightly conical, not attenuated and with no calyptra. Sheath absent.

Ecology: Sites BP-KPB2 and G-KRB

Notes: Trichome width and apical cell characteristics consistent with *O. pseudocurviceps* as discussed by Komárek and Anagnostidis (2005). However, this morphospecies is reported from a reservoir in South Africa (Komárek & Anagnostidis 2005). The occurrence of this morphospecies in brackish water has not been noted previously.

***Oscillatoria rupicola*** Hansgirg (1890) (Figs. 6g – 6i)

Komárek and Anagnostidis (2005): p. 586, Fig. 874 (p. 585)

Description: Field specimens epiphytic on rotting tree branches (Fig. 2b). Trichomes olive-green to bright blue-green, free-living among other algae, straight or slightly curved, isopolar. Cells cylindrical, distinctly shorter than wide, 5.0 μm–6.3 μm wide, 1.3 μm–2.5 μm long, not constricted at the granulated cross wall. Apical cells rounded, not attenuated with no calyptra. Sheath absent.

Ecology: Sites BP-KPB3 and G-KRB

Notes: Previously recorded as subaerophytic on wet rocks, walls (Komárek & Anagnostidis 2005) and periphyton in flowing water from various localities in Uttar Pradesh, India (Kesarwani *et al*. 2015). However, the presence of this morphospecies in brackish or saline water is not well documented.

**Figure 7:**
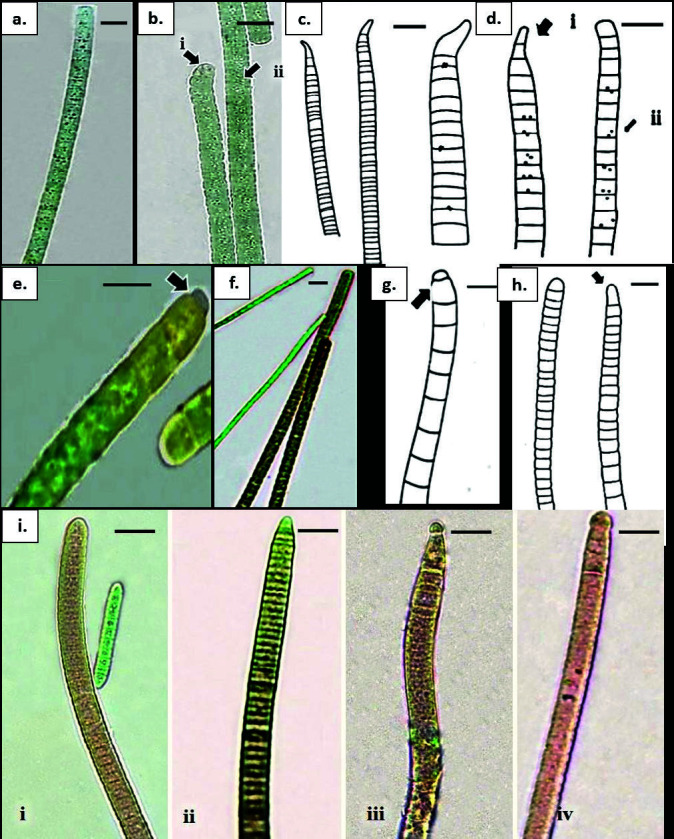
(a) Phormidium cf. bulgaricum. bright blue-green trichome with unconstricted cross walls, heavily granulated cells (arrow), rounded apical cell. (b) *Phormidium formosum*. trichome morphology with nearly isodiametric cells, constricted cross walls, mature trichome with slightly attenuated, conically rounded apical cell (arrow); (c) Phormidium cf. janthiphorum. trichome morphology with nearly isodiametric cells, unconstricted cross walls, mature filament with slightly attenuated, bent, pointed (arrow); (d) Phormidium cf. laetevirens. isodiametric cells, mature trichome with gradually attenuated, conically rounded apical cell [(arrow (i)], cell content granulated [(arrow (ii)]; (e & g) Phormidium cf. subsalsum. mature trichome with rounded apical cell and the presence of calyptra (arrow). (f & h) Phormidium cf. nigroviride. olive-green trichome associated with Geitlerinema cf. tenuius, nearly isodiametric, slightly attenuated with conically rounded apical cell (arrow); (i) *Phormidium uncinatum*. olive green or bright blue-green trichome with distinctly shorter than wide, mature trichome with variation in apical cell shape, (i) widely-rounded, (ii) conically rounded, (iii) conically rounded with calyptra, (iv) widely rounded with calyptra. Scale bars: a and b = 10 μm, c–i = 5 μm.

***Phormidium* cf. *bulgaricum*** (Komárek) Anagnostidis et Komárek (1988) (Fig. 7a)

Komárek and Anagnostidis (2005): p. 442, Fig. 642 (p. 441)

Description: Field specimens epipelic inside PVC water pipe (Fig. 2h) and epiphytic on rotting tree branches (Fig. 2b). Trichomes bright blue-green, long, straight, isopolar. Cells cylindrical, slightly shorter than wide to nearly isodiametric, 4.0 μm–5.0 μm wide, 2.0 μm–6.0 μm long, not constricted at cross walls. Cell content finely granulated. Apical cells rounded, not attenuated with no calyptra. Sheath absent.

Ecology: Sites BP-KSP1 and epiphytic in G-KRB

Notes: Specimens’ differ from *P. bulgaricum* described by Komárek and Anagnostidis (2005) in having slightly narrower (2.5–3.3 (15) μm) and longer cells (1.0–4.0 μm). Previously recorded as halophilic and subaerophytic, mixed with other cyanobacteria in benthic mats (Komárek & Anagnostidis 2005).

***Phormidium formosum*** (Bory ex Gomont) Anagnostidis et Komárek (1988) (Fig. 7b)

Komárek and Anagnostidis (2005): p. 421, Fig. 602 (p. 423)

Description: Field specimens epipelic (Fig. 2d). Trichomes bright blue-green, long, straight, isopolar. Cells cylindrical, slightly shorter than wide or nearly isodiametric, 5.0 μm–6.3 μm wide, 3.5 μm–5.0 μm long, slightly constricted at cross walls. Cell content finely granulated. Apical cells bent or conically rounded, not attenuated with no calyptra. Sheath absent.

Ecology: Sites BP-KPB2, G-KM and BP-KPP.

Notes: This morphospecies is similar morphologically to *P. breve* (Kützing ex Gomont) Anagnostidis et Komárek but differs in having slightly longer cells (1.5 μm–3.0 μm) (Komárek & Anagnostidis 2005). Cosmopolitan with previous records from periphytic and benthic stagnant waters, brackish and saline waters (Komárek & Anagnostidis 2005). Also recorded from wetlands in coastal lagoons in southern Brazil (Martins *et al*. 2012).

***Phormidium* cf. *janthiphorum*** (Fiorini-Mazzetti e Gomont) Elenkin 1949 (Fig. 7c)

Komárek and Anagnostidis (2005): p. 400, Fig. 552 (p. 401)

Description: Field specimens epipelic inside pvc water pipe (Fig. 2h). Trichomes pale blue-green, straight, and actively motile by oscillation movement, isopolar. Cells cylindrical, slightly shorter than wide and nearly isodiametric, 5.0 μm–5.5 μm wide, 4.0 μm–4.5 μm long, not constricted at cross wall. Apical cells pointed to sharply pointed, slightly curved to the ends, and attenuated with no calyptra. Sheath absent.

Ecology: Site BP-KPB1

Notes: This morphospecies similar morphologically to *P. janthiphorum* but differs in having slightly shorter cells [3.3–6.7(7)] μm (Komárek & Anagnostidis 2005). *P. janthiphorum* previously recorded in thermal and warm springs (Komárek & Anagnostidis 2005).

***Phormidium* cf. *laetevirens*** (Crouan ex Gomont) (Fig. 7d)

Komárek and Anagnostidis (2005): p. 415, Figs. 588 and 589 (p. 417)

Description: Field specimens epipelic inside plastic bottle (Fig. 2e). Trichomes bright blue-green, long, straight, isopolar. Cells cylindrical, slightly longer than wide or nearly isodiametric, 3.5 μm–5.0 μm wide, 4.4 μm–5.0 μm long, slightly constricted at cross walls. Cell content finely granulated. Apical cells conically rounded, slightly attenuated with no calyptra. Sheath absent.

Ecology: Sites BP-KSP1 and G-KM.

Notes: *P. laetevirens*, previously known as as *Oscillatoria laetevirens*, is differentiated by the presence of a thin sheath (Saha *et al*. 2007). This specimen differs ecologically as discussed by Komárek and Anagnostidis (2005), who recorded the species as periphyton on stones and rocks from marine and sulphur thermal spring ecosystems and as being widely distributed in Europe.

***Phormidium* cf. *subsalsum*** Gomont 1829 (Figs. 7e & 7g)

Komárek and Anagnostidis (2005): p. 469, Fig. 698 (p. 471)

Description: Field specimens epipelic (Fig. 2d). Trichomes yellowish or bright blue-green, straight, heteropolar. Cells cylindrical, nearly isodiametric, 4.5 μm–5.5 μm wide, 4.0 μm–6.5 μm long, not constricted at cross wall. Apical cells rounded, not attenuated with the present of calyptra at one end. Sheath absent.

Ecology: Sites BP-KPB3, BP-KP2 and BP-KPP

Notes: Specimen differs from *P. subsalsum* described by Komárek and Anagnostidis (2005) in having slightly longer trichome (6.0 μm–7.0 μm) and differences in apical cell morphology (attenuated, slightly bent, spirally coiled or hooked). Previously recorded from brackish water in coastal Norway (Komárek & Anagnostidis 2005).

***Phormidium* cf. *nigroviride*** Thwaites in Harvey (1846) (Figs. 7f & 7h)

Komárek and Anagnostidis (2005): p. 579, Fig. 861 (p. 580)

Description: Field specimens periphytic on plastic food containers (Fig. 2f). Trichomes dark olive-green to brown, long, straight, isopolar. Cells cylindrical, shorter than wide and nearly isodiametric, 5.0 μm–6.3 μm wide, (2.0)2.5–5.0 μm long, slightly constricted at cross walls. Apical cells rounded or conically rounded and slightly attenuated towards the end with no calyptra. Sheath absent.

Ecology: Sites BP-KSB2, BP-KBAH and BP-KPP

Notes: Specimen differs from *O. nigroviridis* described by Komárek and Anagnostidis (2008) in slightly narrower trichome, 7.0–12.0 (13.0) μm. Previously recorded in marine ecosystems in the Mediterranean as epilithic and widely distributed in warm water column (Komárek & Anagnostidis 2005). Also recorded from estuaries as epipelic in a mangrove swamp in Nigeria (Essien & Ubom 2003).

***Phormidium uncinatum*** Gomont ex Gomont 1892 (Fig. 7i)

Komárek and Anagnostidis (2005): p. 481, Fig. 719 (p. 482)

Description: Field specimens epiphytic on pneumatophores (Fig. 2a). Trichomes olive-green or bright blue-green, long, straight or slightly bent, actively motile, heteropolar. Cells cylindrical, distinctly shorter than wide, 6.4 μm–8.8 **μm** wide, 2.5 μm–3.2 μm long, not constricted at cross wall. Apical cells obtuse, rounded, conically rounded or slightly pointed, not attenuated with calyptra. Sheath absent.

Ecology: Sites BP-KBAH and G-KRB

Notes: Previously recorded as a cosmopolitan species from rocks, stones, wood and other substrata, and rarely from moist soils and brackish swamps (Komárek & Anagnostidis 2005).

### Family Coleofasciculaceae

**Figure 8:**
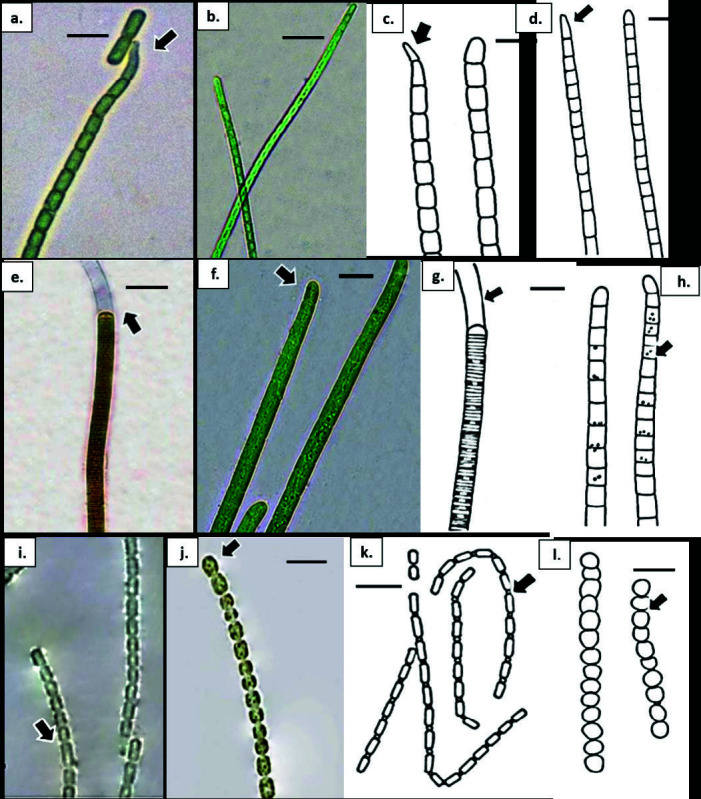
(a) & (c) Geitlerinema attenuatum; (a) bright blue-green, mature trichome with slightly constricted cross walls; (c) attenuated, bent with pointed apical cell (arrow). (b) & (d) Geitlerinema cf. tenuius; (b) apical cell conically rounded; (d) pointed apical cell (arrow). (e) & (g) Leptolyngbya cf. pauciramosa; (e) trichome morphology with distinctly shorter than wide cells and rounded apical cell (arrow); (g) colourless sheath (arrow); (f) & (h) Leptolyngbya subuliformis; (f) mature trichome with slightly attenuated, conically rounded apical cell (arrow); h. bright blue-green trichome entangled with nearly isodiametric cells, slightly constricted at cross walls with finely granulated cell (arrow); (i) & (k) Pseudanabaena cf. amphigranulata. i. trichome pale blue-green, cylindrical towards apical cells (arrow); (k) trichomes with cells longer than wide, present of aerotopes (arrow); (j) & (l) Pseudanabaena sp. (j) dark olive-green trichome, distinctly constricted, rounded apical cell (arrow); (l) visible aerotopes (arrow). Scale bars: (d) & (f) = 5 μm; a–c, e, g–l = 10 μm.

***Geitlerinema attenuatum*** (Voronichin) Anagnostidis 2001 (Figs. 8a & 8c)

Komárek and Anagnostidis (2005): p. 131

Description: Field specimens epipelic inside PVC water pipe (Fig. 2h) and periphytic on rotting tree branches (Fig. 2b). Trichomes pale blue-green or bright blue-green, straight, motile with slowly oscillation movement, isopolar. Cells cylindrical, distinctly longer than wide, 3.0 μm–3.8 μm wide, 5.0 μm–7.5 μm long, slightly constricted at cross walls. Apical cells conical or pointed, slightly bent, attenuated with no calyptra. Sheath absent.

Ecology: Sites BP-KPB, BP-KBAH and G-KM

Notes: The motility of the trichome and mat forming characteristics suggest placement in *Geitlerinema* rather than *Jaaginema*, which has immotile trichomes and occurs mainly in clusters (Komárek & Anagnostidis 2005). This morphospecies recorded as halophilic and epiphytic on other algae and cyanoprokaryotes in saline lakes (Komárek & Anagnostidis 2005).

***Geitlerinema* cf*. tenuius*** (Stockmayer) Anagnostidis (2001) (Figs. 8b & 8d)

Komárek and Anagnostidis (2005): p. 129

Description: Field specimens epipelic inside plastic bottle (Fig. 2e). Trichomes bright blue-green, straight and entangled, isopolar. Cells cylindrical, slightly longer than wide, 1.8 μm–2.5 μm wide, 3.2 μm–6.2 μm long, slightly constricted at cross walls. Apical cells conically rounded or pointed, not attenuated with no calyptra. Sheath absent.

Ecology: Sites BP-KPB4 and BP-KSB1

Notes: Specimens differ from *G. tenuius* described by Komárek and Anagnostidis (2005) in having the morphology of the cross wall not constricted. *G. tenuius* is previously recorded from moist soils, shallow ditches and paddy fields (Komárek & Anagnostidis 2005). However, the presence of this morphospecies in brackish or saline water is not well documented.

### Family Leptolyngbyaceae

***Leptolyngbya* cf. *pauciramosa*** (Anisimova) Anagnostidis et Komárek (1988) (Figs. 8e and 8g)

Komárek and Anagnostidis (2005): p. 200, Fig. 247 (p. 207)

Description: Field specimens epipelic on tip of pneumatophore (Fig. 2a). Filaments dark olive-green, long, straight, isopolar. Cells cylindrical, distinctly shorter than wide, 3.0 μm–3.5 μm wide, 0.5 μm–1.25 μm long, not constricted at cross walls. Apical cells rounded, not attenuated with no calyptra. Sheath thin, hyaline.

Ecology: Site G-KM

Notes: Specimens’ consistent with the width of *L. pauciramosa* described by Komárek and Anagnostidis (2005), but no information for length available to confirm the determination. *L. pauciramosa* is previously recorded from saline, mineral lakes and inland saline lakes in Ukraine (Komárek & Anagnostidis 2005).

***Leptolyngbya subuliforme*** (Kützing ex Gomont) Anagnostidis et Komárek (1988) **(**Figs. 8f and 8h)

Komárek and Anagnostidis (2005): p. 408, Fig. 573 (p. 407)

Description: Field specimens epipelic inside plastic bottle (Fig. 2e). Trichomes bright blue-green, straight, isopolar. Cells cylindrical, slightly longer than wide or nearly isodiametric, 5.0 μm–6.4 μm wide, 5.0 μm–7.5 μm long, slightly constricted at cross walls. Cell contents finely granulated. Apical cells conical-rounded, gradually attenuated with no calyptra. Sheath absent.

Ecology: Site G-KM

Notes: *P. subuliforme* cells, as described by Komárek and Anagnostidis (2005), are not constricted at cross wall. According to Strunecký *et al*. (2013), *P. subuliforme* is one of the species revised to belong to the new genus, *Kamptonema*, but their position needs to be confirmed by further studies and molecular assessment. Previously recorded as widely distributed, occurring in marine ecosystem and on coastal rocks as well as from brackish waters.

### Family Pseudanabaenaceae

***Pseudanabaena* cf. *amphigranulata*** (Van Goor) Anagnostidis 2001 (Figs. 8i and 8k)

Komárek and Anagnostidis (2005): p. 86, Fig. 66 (p. 87)

Description: Field specimens epiphytic on rotting tree branches (Fig. 2b). Trichomes olive-green, long, straight or bent with 20–30 cells, isopolar. Cells nearly barrel-shaped to cylindrical, longer than wide to almost isodiametric, 1.2 μm–1.8 μm wide, 2.2 μm–2.8 μm long, distinctly constricted at cross walls with one large aerotope at each cross wall. Apical cells rounded, not attenuated with no calyptra. Sheath absent.

Ecology: Sites BP-KPB1, BP-KPB3 and BP-KM

Notes: Specimen differs by having shorter cell [(2–5(7)] μm compared to *P. amphigranulata* described by Komárek and Anagnostidis (2005). Previously recorded from various fresh and brackish water bodies, and benthic habitats (Komárek & Anagnostidis 2005).

***Pseudanabaena* sp**. (Figs. 8j and 8l)

Komárek and Anagnostidis (2005): p. 70

Description: Field specimens epiphytic on rotting tree branches (Fig. 2b). Trichomes olive-green, straight or slightly bent with 5–8 cells, isopolar. Cells slightly barrel-shape, almost isodiametric, 2.0 μm–3.0 μm wide, 2.0 μm–3.8 μm long, distinctly constricted at cross walls, sometimes with aerotopes. Apical cells widely rounded, not attenuated with no calyptra. Sheath absent.

Ecology: Sites BP-KPB1, BP-KPB3, BP-KP1, BP-KBAH and BP-KPP

Notes: Specimens similar morphologically to *P. minima* described by Komárek and Anagnostidis (2005) but differ by only having wider cell, 1.3 μm–2.5 μm wide, while *P. minima* has no aerotopes.

### Family Spirulinaceae

**Figure 9:**
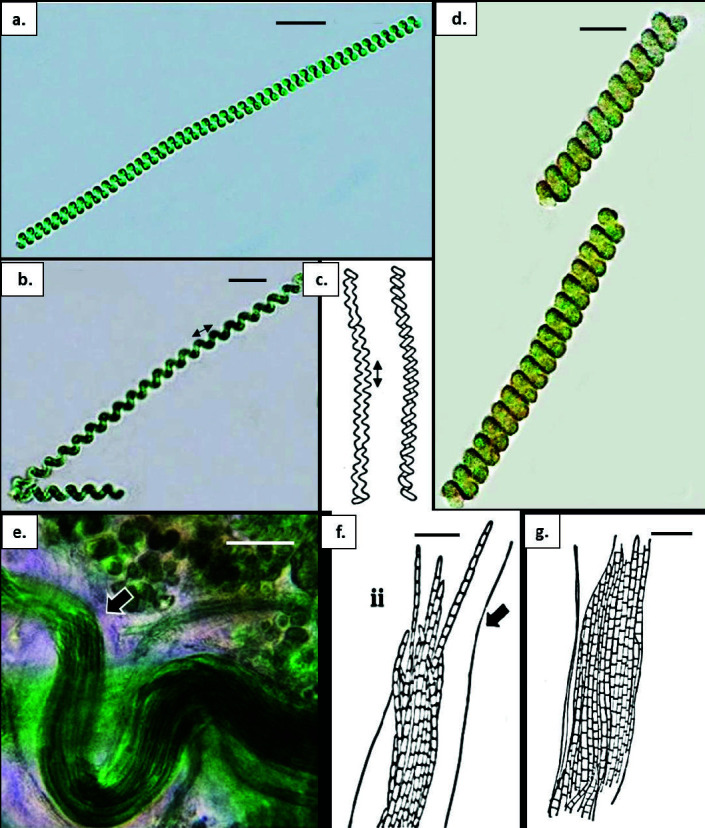
(a) *Spirulina* cf. *labyrinthiformis*. a. trichomes bright blue-green, straight or slightly curved, densely screw-like coiled, tightly joined; (b) & (c). *Spirulina* cf. *meneghiniana*. trichome loosely coiled, distance between coil clearly seen (arrow); (d) *Spirulina* cf. *robusta*. trichomes olive-green to reddish brown, straight or slightly curved, dense screw-like coiled, tightly joined, solitary among other cyanobacteria; (e) & (f) *Trichocoleus tenerrimus;* (e) filaments aggregated among other microalgae; f. (ii) bundle of trichomes assembled in one single thick sheath, open, hyaline (arrow); (g) *Trichocoleus* cf. *voukii*. trichomes cylindrical in one single sheath, open, hyaline (arrow). Scale bars: b–d = 5 μm; a, e, f & g = 20 μm.

***Spirulina* cf. *labyrinthiformis*** Kützing ex Gomont 1892 (Fig. 9a)

Komárek and Anagnostidis (2005): p.146, Fig. 171 (p. 145)

Description: Field specimens epipelic (Fig. 2d). Trichomes bright blue-green, free-living, evenly spiral coiled to each other, distance between coils 1.5 μm–1.8 μm, actively motile with right-handed screw-like rotation, isopolar. Cells shorter than wide, 3.0 μm–3.5 μm wide, 1.5 μm–1.8 μm long, not constricted at cross walls. Apical cells rounded, not attenuated. Sheath absent.

Ecology: Sites BP-KPB1, BP-KPB3 and G-KM

Notes: Specimen differs from *S. labyrinthiformis* described by Komárek and Anagnostidis (2005) in having wider cells [(1.5)2.0–2.7(3.0)]. Previously recorded as a cosmopolitan morphospecies, found in brackish and saline waters and thermal springs. Naskar (2008) documented this morphospecies in brackish water of North 24-Parganas district, West Bengal, tolerating a salinity range of 3.4 g/L–8.4 g/L.

***Spirulina* cf. *meneghiniana*** Zanardini ex Gomont 1892 (Figs. 9b & 9c)

Komárek and Anagnostidis (2005): p. 147, Fig. 172 (p. 149)

Description: Field specimens epipelic (Fig. 2d). Trichomes bright blue-green, free-living, solitary, straight or slightly bent, loosely screw-like coiled to each other, distance between coils 4.0 μm–5.0 μm, actively motile with right-handed screw-like rotation. Isopolar. Cells shorter than wide, 1.2 μm–2.0 μm long with 3.8 μm–5.0 μm wide, not constricted at cross wall. Apical cells rounded and not attenuated. Sheath absent.

Ecology: Site BP-KPB2

Notes: Specimens vary in slightly wider cells [(1.2–1.8(2.0)] μm. Previously recorded in brackish water wetlands of North 24-Parganas, west Bengal, India (Naskar *et al*. 2008).

***Spirulina* cf. *robusta*** (Welsh) 1965 (Fig. 9d)

Komárek and Anagnostidis (2005): p. 150, Fig. 177 (p. 153)

Description: Field specimens epiphytic on rotting tree branch (Fig. 2b). Trichomes reddish brown to blackish, slimy, free-living, solitary, mixed with other cyanobacteria, straight or slightly bent, tightly coiled to each other, distance between coils 1.5 μm–1.8 μm, motile with right-handed screw-like rotation, isopolar. Cells shorter than wide, 3.0 μm–4.5 μm wide, 1.0 μm–1.5 μm long, not constricted at cross walls. Apical cells rounded and not attenuated. Sheath absent.

Ecology: Sites BP-KPB4 and BP-KSB2

Notes: Specimens vary in having slightly wider cells (2.0 μm–3.0 μm) and shorter coil distance [2.0–2.7 (–5)] μm to that suggested by Komárek and Anagnostidis (2005). Mat characteristics and motility have not been well described for the morphospecies that hinders comparison with the present specimens. Previously recorded from freshwater or slightly saline water, in south-east Asian countries, India and the Philippines (Komárek & Anagnostidis 2005).

### Family Trichocoleusaceae

***Trichocoleus tenerrimus*** (Gomont) Anagnostidis (2001) (Figs. 9e & 9f)

Komárek and Anagnostidis (2005): p. 316, Fig. 422 (p. 313)

Description: Field specimens epiphytic on rotting tree branches (Fig. 2b). Filaments bright blue-green, entangled, multiple trichomes in single sheath, isopolar. Cells cylindrical, distinctly longer than wide, 1.0 μm–2.2 μm wide, 3.2 μm–4.2 μm long, not constricted at cross walls. Apical cells conically rounded, not attenuated with no calyptra. Sheath thick, hyaline, open or closed at the ends.

Ecology: Site BP-KSP1

Notes: Previously recorded in marine ecosystems, on rocks, rock pools and algae in littoral and supralittoral zones. Komárek and Anagnostidis (2005) documented *T. tenerrimus* to occur on roots of mangroves.

***Trichocoleus* cf. *voukii*** (Frémy ex Frémy) Anagnostidis (2001) (Fig. 9g)

Komárek and Anagnostidis (2005): p. 316, Fig. 423 (p. 313)

Description: Field specimens epipelic (Fig. 2d) and periphytic on plastic bottle (Fig. 2e). Filaments green or bright blue-green, entangled with other cyanobacteria, isopolar. Cells cylindrical, distinctly longer than wide, 1.5 μm–2.5 μm wide, 5.5 μm–10.4 μm long, not constricted at cross walls. Apical cells conically rounded, not attenuated with no calyptra. Sheath thick, hyaline.

Ecology: Site BP-KP1

Notes: Specimens differ from *T. voukii* described by Komárek and Anagnostidis (2005) in having slightly wider trichome, 1.6 μm–1.8 μm, and slightly longer cell, 3.2 μm–9.0 μm. Previously recorded in marine ecosystems from the Mediterranean coast of France (Komárek & Anagnostidis 2005).

## DISCUSSION

### Cyanobacteria in Balik Pulau and Gurney Mangrove Ecosystems

The 33 morphospecies identified in this study provide new records of periphytic cyanobacteria occurring in Malaysian mangrove ecosystems. The morphospecies are representatives of the groups Chroococcales, Chroococcidiopsidales, Nostocales, Oscillatoriales, Pleurocapsales, Spirulinales and Synechococcales.

Oscillatoriales was the most widespread group recorded. The dominance of the group has also been noted in previous studies in tropical mangroves (Ram & Paul 2021; Ram & Shamina 2017; Branco *et al*. 2003; Nogueira & Ferreira-Correia 2001). The ability of members of this group to tolerate considerable fluctuation in salinity, temperature, water volume, light intensity and UV radiation enables their successful colonization in this extreme ecosystem (Mandal & Rath 2015). Members of this group, for example in the genera *Phormidium* and *Oscillatoria*, are able to survive under osmotic stress and tolerate a wide range of salinity fluctuation (Mandal & Rath 2015). Mangrove salinity is a major environmental factor that changes rapidly through the tidal cycle, along with temperature changes. Salinity generally increases during the flooding tide and decreases during the ebbing tide, although desiccation of exposed surfaces can conversely lead to increased osmotic stress locally. High temperature will increase evaporation rate thus further increasing salinity. The presence of organic osmoregulatory solutes in members of the group enables them to maintain their intracellular ionic concentration at low levels despite constant inwards diffusion of K^+^ and Cl^−^ ions from the environment (Mandal & Rath 2015).

Seven of the 33 morphospecies recorded in this study were motile. Motility rates differed between them, with six morphospecies (*O. pseudocurviceps, P*. cf. *janthiphorum, P. uncinatum, S*. cf. *labyrinthiformis, S. meneghiniana* and *S*. cf. *robusta*) having rapid gliding movement, while one morphospecies (*G. attenuatum*) showed a slow oscillation movement. Downwards and upwards movements of motile Oscillatoriales allow migration from microbial mat surfaces into soft sediments, to avoid long-term exposure to high levels of ultra-violet radiation (Bagmi *et al*. 2007). High exposure to solar radiation is common in areas within tropical mangroves and can have an adverse impact on growth, survival, pigmentation, orientation, metabolism and photosynthesis in cyanobacteria (Xue *et al*. 2005). Motility could also enable these morphospecies to reposition buried trichomes to the surface of the sediment following disturbance during the tidal cycle.

Heterocytous cyanobacteria were the group least recorded in this study compared to unicellular/colonial or non-heterocytous filamentous types. Only three genera were recorded, *Anabaena, Desmonostoc* and *Nostoc*, all representing the order Nostocales. Among these, *Anabaena* sp. and *Nostoc* sp. were only recorded in culture. There may be two possible explanations for this observation - both morphospecies may have been present in very low abundance, hindering their identification in fresh sample material, or they could derive from wind-dispersed spores from the nearby terrestrial environment.

Most studies of cyanobacteria in tropical mangroves have reported low occurrence of heterocytous species (Ram & Paul 2021; Ram & Shamina 2017; Alvarenga *et al*. 2015). This may be attributed to the likely high levels of nitrogen and the instability of their macroscopic growth form in this harsh environment. Low nitrogen levels in the environment favour the occurrence of nitrogen-fixing cyanobacteria and this can be reflected in the number of heterocytous groups present (Sakthivel & Kathiresan 2013). Poor adaptation and weak resistance of heterocytous cyanobacteria towards water disturbance may break the weaker connections between the heterocyte and vegetative cells (Stal & Krumbein 1985; Stal 1995).

The most common macroscopic growth form noted in this study was that of mats. Cyanobacterial mats collected from both sampling areas were usually dominated by more than one morphospecies, contradicting the conclusion of Stal (1995) who noted that many cyanobacterial mat types are dominated by a single species. In the present study, the co-occurrence of various species in the mat may relate to the instability of the environment. We suggest that the occurrence of different morphospecies together allows interaction and enables sharing of resources, thereby enhancing survival of the community. The presence of different morphospecies with similar functions, or morphospecies with different functions, could improve overall tolerance of the rapidly changing environment.

Among the cyanobacteria collected, three morphospecies—*Aphanocapsa* cf. *concharum, Xenococcus* cf. *pallidus* and *O. pseudocurviceps*—are rare and poorly-known morphospecies worldwide. Two of these morphospecies, *A*. cf. *concharum* and *O. pseudocurviceps*, were found both in Balik Pulau and Gurney. *X. pallidus* has previously been collected from the marine ecosystem (Komárek & Anagnostidis 1998), but here was only recorded in the mangrove area, and only at the Balik Pulau sampling site.

### Previous Records of the Morphospecies Encountered

Brazil and India are the current leaders in cyanobacterial studies in mangrove ecosystems (Alvarenga *et al*. 2015). However, surveys of cyanobacterial diversity in mangroves have also been conducted in a few other regions of the world, including Egypt, Mexico, Nigeria, Saudi Arabia, South Africa and Tanzania (Alvarenga *et al*. 2015). These studies provide baseline data for cyanobacterial diversity in different mangrove habitats. Several taxa (*Aphanocapsa litoralis*, *Chroococcus minutus*, *Xenococcus* cf. *schousboei*, *Lyngbya* cf. *aestuarii*, *Phormidium* cf. *nigroviride*, *Spirulina* cf. *labyrinthiformis*, *Spirulina meneghiniana*), appear to be cosmopolitan in mangrove ecosystems (Dor *et al*. 1984; Hussain & Khoja 1993; Selvakumar & Sundararaman 2001; Nedumaran *et al*. 2008; Pérez-Estrada *et al*. 2012; Silambarasan *et al*. 2012; Sakthivel & Kathiresan 2013).

*Chroococcus minutus* in this study was epiphytic on rotting tree branches from Balik Pulau and on a pneumatophore in Gurney, while *X. schousboei* was recorded from rotting tree branches in both areas. Submerged parts of mangrove trees, branches and pneumatophores potentially serve as habitat for epifloral and faunal communities (Sundararaman *et al*. 2007; Mohamed & Al-Shehri 2015). However, previous records of both *C. minutus* and *X. schousboei* are from sediment (Hussain & Khoja 1993; Branco *et al*. 2003; Sakthivel & Kathiresan 2013; Mohamed & Al-Shehri 2015). The occurrence of these two morphospecies appears not to be entirely dependent on attachment structure, allowing them to be more widespread on a wider range of available substrata.

*Lyngbya* cf. *aestuarii* was found growing on diverse substrates in this study. Similar observations have been reported from mangroves in India (Sakthivel & Kathiresan 2013). Members of the genus *Lyngbya*, together with *Oscillatoria, Phormidium* and *Microcoleus*, are widespread in mangrove areas (Sundararaman *et al*. 2007). *Lyngbya aestuarii* was previously recorded as a dominant mat-forming cyanobacteria in hypersaline Laguna Guerrero Negro, Mexico (Bagmi *et al*. 2007). The widespread occurrence of this species may be attributed to the presence of the sheath, which helps in binding cells to support their structure and attachment to sand grains (Rajeev *et al*. 2013; Rossi & De Philippis 2015). The sheath pigment, scytonemin, also gives effective protection from excessive ultra-violet exposure (Rastogi & Incharoensakdi 2014), reduces dehydration (Rossi & De Philippis 2015) and acts as a structural defence against predators (Camacho & Thacker 2006).

*Phormidium* cf. *nigroviride* was found mainly on sediment inside plastic food containers. Similar morphospecies have been recorded previously but differ in their type of attachment, occurring on pneumatophores and on sediment (Sakthivel & Kathiresan 2013; Mohamed & Al-Shehri 2015). In this study, *P*. cf. *nigroviridis* was collected at exposed sampling sites and near to aquaculture farms and residential areas, which had high abundance of plastic containers and debris. This species may not require a specific substrate for attachment but rather display a more opportunistic behaviour that allows it to thrive at such disturbed sites.

*Spirulina* cf. *labyrinthiformis* and *S. meneghiniana* were found epipelic on sediments. *S. labyrinthiformis* was previously recorded as ubiquitous in mangrove areas (Hussain & Khoja 1993; Lugomela *et al*. 2001; Sakthivel & Kathiresan 2013; Ahmad *et al*. 2016). Sakthivel and Kathiresan (2013) reported this morphospecies from all three sampling sites examined in a study on the south-east coast of India, Pichavaram, Porto Novo and Mudasal Odai. A similar pattern observed in the present study further supports the ubiquity of these species.

Comparison of the morphospecies recorded in this study with those from other mangrove ecosystems worldwide revealed only seven morphospecies to be common. The present study raises the possibility of the cyanobacterial microflora of Penang mangroves being distinct from other assemblages around the world. Limitations in the identification keys currently available, and particularly their reliance on material derived from temperate regions, could provide a significant source of confusion as well as the loss of important information on endemic and rare species occurring in tropical regions. There is clearly a need to further develop taxonomic knowledge and keys to fully assess the diversity of this group. Robust identification of many tropical cyanobacteria requires further integrated study of their morphological, ecological and molecular characteristics in Malaysian and other mangrove ecosystems.

Land conversion is already extensive in both mangrove areas surveyed in this study. Hundreds of hectares in the mangrove forest area of Pulau Betung have been developed to provide shrimp ponds and residential areas. Large parts of the Gurney Drive mangrove area have also undergone reclamation and with the development of roads and buildings. These disturbances are reflected by the dominance of the Oscillatorialles in our data. Members of the Oscillatorialles are excellent indicators of eutrophication related to alterations in land use (Chaurasia 2015), as observed at this study site. The highly enriched wastewater from commercial aquaculture is likely to have triggered a rapid response by these cyanobacteria, leading to their dominance over heterocytous species. Floristic studies can provide important baseline information not only on the diversity but also on the potential use of cyanobacteria in monitoring the health of mangroves in other mangrove areas around Malaysia and more widely.

## Figures and Tables

**Table 1:** Summary description of the sampling sites. Degree of shading was estimated based on Harding *et al*. (2009), including the categories: unshaded (0%–30%), partly shaded (30%–60%), shaded (>80%).

Sampling site	Code used	GPS coordinates	Habitat description	
	
	Balik Pulau (BP)		Nearby area	Degree of shading
Kampung Pulau Betung (KPB)	BP-KPB1	N5o18′28.4″ E100o11′44.7″	Sungai Pulau Betung	Partly shaded
	BP-KPB2	N5o18′23.3″ E100o12′02.4″	Residential area and shrimp aquaculture pond	Partly shaded
	BP-KPB3	N5o18′33.3″ E100o11′52.4″	Residential area and shrimp aquaculture pond	Partly shaded
	BP-KPB4	N5o18′50.4″ E100o11′52.3″	In mangrove forest	Unshaded
Kampung Perlis (KP)	BP-KP1	N5o18′59.2″ E100o11′52.5″	Vegetable farm	Shaded
	BP-KP2	N5o19′24.7″ E100o11′48.7″	Abandoned aquaculture pond	Shaded
Kampung Sungai Burung (KSB)	BP-KSB1	N5o19′40.6″ E100o11′48.3″	Sungai Nipah	Shaded
	BP-KSB2	N5o20′13.6″ E100o11′46.9″	Fishing jetty and residential area	Unshaded
Kampung Jalan Baru (KJB)	BP-KJB	N5o21′01.2″ E100o11′44.5″	Sungai Kongsi and residential area	Unshaded
Kampung Bagan Air Hitam (KBAH)	BP-KBAH	N5o21′42.1″ E100o11′30.9″	Aquaculture pond	Shaded
Kampung Permatang Pasir (KPP)	BP-KPP	N5o22′14.4″ E100o11′27.4″	Aquaculture pond	Unshaded
Kuala Sungai Pinang (KSP)	BP-KSP1	N5o22′34.5″ E100o11′28.5″	Between two aquaculture ponds	Partly shaded
	BP-KSP2	N5o23′28.1″ E100o11′42.6″	Residential area and restaurant	Partly shaded

	Gurney (G)			

Kampung Masjid	G-KM	N5o26′34.3″ E100o18′28.9″	Residential area, restaurant and water discharge	Unshaded
Kampung Raya Baharu	G-KRB	N5o26′37.2″ E100o18′28.4″	Residential area	Shaded

**Table 2:** List of cyanobacteria identified from mangroves sampled Balik Pulau and Gurney.

Family	Species	Field		Culture			

		Balik Pulau	Gurney	Balik Pulau		Gurney	

				U	M	U	M
Chroococcaceae	*Chroococcus minutus*	+	+				
Microcystaceae	*Microcystis halophilia*	+		+			
Merismopediaceae	*Aphanocapsa* cf. *concharum*	++					
Cyanobacteriaceae	*Cyanobacterium* cf. *cedrodum*	++					
Chroococcidiopsidacea	*Chroococcidiopsis* cf. *thermalis*	++					
Hyellaceae	*Myxosarcina* cf. *gloeocapsoides*	++	+	+			
Xenococcaceae	*Xenococcus* cf. *pallidus*	++					
	*Xenococcus* cf. *schousboei*	+	++				
	*Anabaena* sp.			+			
Nostocaceae	Demonostoc muscorum	+		+			
	*Nostoc* sp.			+			
	*Lyngbya* cf. *aestuarii*	+					
	*Lyngbya* cf. *salina*	++					
	Oscillatoria pseudocurviceps	+	+	+		+	
	Oscillatoria rupicola	++	+				
	*Phormidium* cf. *bulgaricum*	+	+				
Oscillatoriaceae	Phormidium formosum	+	+				
	*Phormidium* cf. *janthiphorum*	++					
	*Phormidium* cf. *laetevirens*	++	+	+			
	*Phormidium* cf. subsalsum	++					
	*Phormidium* cf. *nigroviride*	++			+		
	Phormidium uncinatum	++	++				
Coleofasciculaceae	*Geitlerinema attenuatum*	++	++	+		+	
	*Geitlerinema* cf. *tenuius*	+					
Leptolyngbyaceae	*Leptolyngbya* cf. *pauciramosa*		++				
	Leptolyngbya subuliformis	+	++				+
Pseudanabaenaceae	*Pseudanabaena* cf. *amphigranulata*	++	++				
	*Pseudanabaena* sp.	++					
	*Spirulina* cf. *labyrinthiformis*	++	++				
Spirulinaceae	Spirulina meneghiniana	+					
	*Spirulina* cf. *robusta*	+					
Trichocoleusaceae	*Trichocoleus tenerrimus*	++			+		
	*Trichocoleus* cf. *voukii*	+					

*Notes:* U = unialgal cultures; M = mixed cultures; (+) = strain presence; (++) = dominant strains.
